# Right Internal Mammary Artery Occlusion in a Patient With Failed Left Internal Mammary Artery Coronary Artery Bypass Graft Surgery Post a Laparoscopic Appendectomy for Acute Appendicitis

**DOI:** 10.7759/cureus.32343

**Published:** 2022-12-09

**Authors:** Tebianne M Abubaker, Karl M Richardson, Cody J Cox, Fan Ye

**Affiliations:** 1 Internal Medicine, Wake Forest School of Medicine, Winston-Salem, USA; 2 Cardiology, Wake Forest School of Medicine, Winston-Salem, USA; 3 Anesthesiology, Wake Forest School of Medicine, Winston-Salem, USA

**Keywords:** left anterior descending artery, cardio vascular disease, coronary artery bypass grafting(cabg), internal mammary artery, graft

## Abstract

Patients who present with acute myocardial infarction are often urgently evaluated for possible revascularization via coronary artery bypass graft surgery (CABG), percutaneous coronary intervention (PCI), or medical therapy alone. CABG has been shown to provide symptomatic relief as well as increased long-term survival for patients with multivessel coronary artery disease (CAD). Though venous grafts can be used to revascularize the ischemic territory, long-term patency is most successful when using pedicled coronary grafts such as the left internal mammary artery (LIMA) or right internal mammary artery (RIMA) graft. Only a fraction of patients who undergo a RIMA or LIMA will occlude their graft, and mid-graft lesions presumed secondary to atherosclerosis are even rare. For our case report, we evaluate a 72-year-old female who has had a very rare acute coronary occlusion of her mid-RIMA graft resulting in an acute apical left ventricular infarct. A heart catheterization confirmed a 100% thrombotic occlusion of the mid-RIMA-LAD, which was stented with a 2.5 x 20 mm drug-eluting stent.

## Introduction

Coronary arteries are the heart’s main source of oxygenated blood. Atherosclerotic plaques that can form stenosis of coronary arteries can result from risk factors, including hypertension, hyperlipidemia, diabetes, and smoking, amongst others. The use of either internal mammary arteries has become the gold standard for revascularization of proximally stenotic LADs worldwide [1,2]. The use of this technique has led to improved survival, decreased reinfarction, and less need for further PCI or redo coronary bypass [3,4]. While atherosclerosis-mediated thrombotic occlusion of native coronaries is common, unstable atherosclerosis and resultant acute coronary syndromes in IMA grafts are very rare. 

## Case presentation

**Figure 1:**
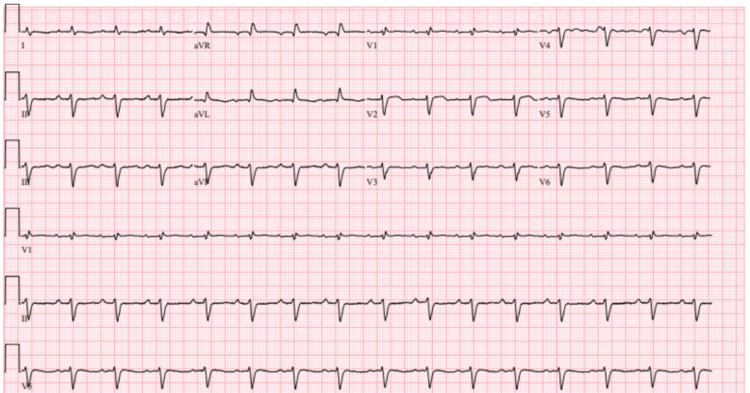
EKG on presentation: sinus rhythm, borderline right bundle branch block, left anterior fascicular block

Our patient is a 72-year-old female with past medical history (PMH) of CAD status post single vessel CABG (LAD-LIMA 2010) with a redo single vessel CABG on 3/19/2021 (RIMA-LAD due to atretic LIMA graft), hypertension, hyperlipidemia, non-small cell lung cancer status post wedge resection in 2009 (no chemotherapy or radiation required). Her cardiology-relevant medications included aspirin 81 mg once daily, ezetimibe 10 mg once daily, evolocumab 140mg/mL injection every 14 days, and clopidogrel 75 mg once daily. Most recent A1c was 5.8%, LDL 29mg/dL, total cholesterol 139 mg/dL, triglycerides 116 mg/dL, and HDL 93 mg/dL. The lipid panel was all within normal limits during this hospitalization. She also had a normal-thyroid stimulating hormone level of 3.34uiu/ml. Of note, she has severe hypercholesterolemia with an elevated low-density lipoprotein cholesterol (LDL-C), for which she has tried several statins and was most recently on evolocumab and ezetimibe; there is a concern for familial hypercholesterolemia as the etiology. She presented to the emergency department (ED) with abdominal pain, nausea, and vomiting in the setting of acute appendicitis and subsequently underwent laparoscopic appendectomy. Postoperatively, on the same day, she started developing chest pain, dyspnea, and nausea. She was afebrile with regular heart rate and respiratory rate, and she was normotensive. Troponin level at the time of draw was 43,118 pg/mL, and EKG showed sinus rhythm with first-degree AV block, right bundle branch block, and dynamic lateral T wave inversions that were concerning for ischemia. She was started on a heparin drip, given aspirin 325mg, and administered sublingual nitroglycerin in the surgical intensive care unit with some symptomatic relief. Her troponin levels decreased to 29,815 on repeat evaluation. Figure 1 shows an EKG that was done at the onset of symptoms. 

**Figure 2:**
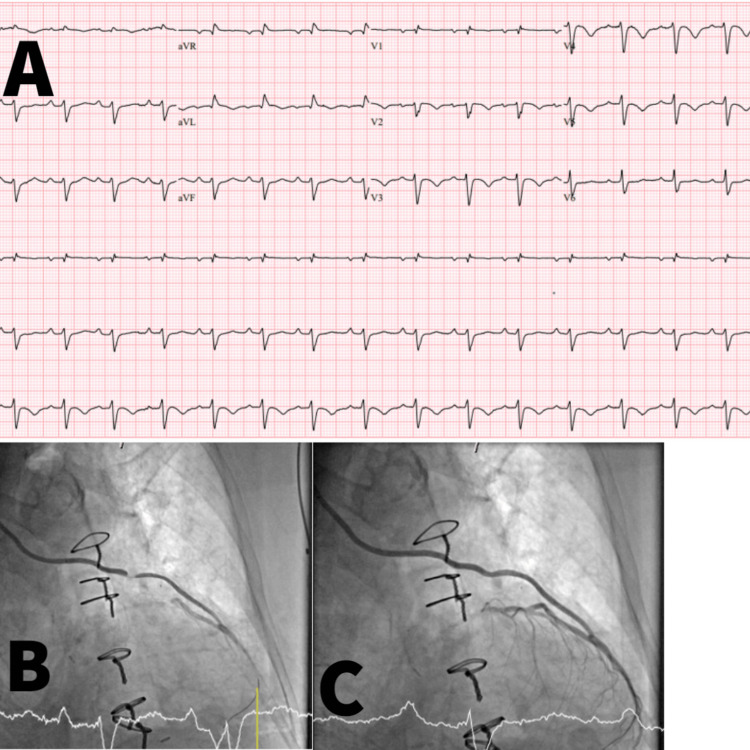
EKG post-op: sinus rhythm, borderline right bundle branch block, left anterior fascicular block, inverted T waves anterolateral precordial leads. Cardiac catheterization showed it was 100% occluded in the body of the right internal mammary artery (B), which was stented with a 2.5x20 Synergy drug-eluting stent (C).

Despite treatment, her chest pain returned with new onset radiation into the jaw bilaterally, at which point she was urgently taken to the catheterization lab. Coronary angiography revealed a persistent 100% occlusion of the proximal LAD new 100% occlusion of the mid-RIMA-LAD graft, in addition to moderate non-obstructive disease in the left circumflex and right coronary artery. After first crossing with a wire, the acute RIMA lesion was eccentric, focal, and appeared to be thrombotic. Transthoracic echocardiogram showed a left ventricular ejection fraction of 35%-40%, akinesis of the LAD territory, including the left ventricular apex. While in the catheterization laboratory, she underwent PCI with a 2.0 x 20 mm drug-eluting stent to the mid-RIMA lesion. After the procedure, she remained free of chest pain; she was monitored via continuous telemetry and daily labs by the cardiology team. Upon discharge, she was started on ticagrelor 90 mg twice a day in addition to her home aspirin to reduce the recurrence of events and improve mortality. For cardiovascular risk reduction, she was continued on ezetimibe 10 mg daily and started on metoprolol succinate 50 mg daily along with losartan 12.5 mg daily. She has since followed up with cardiology and has remained chest pain-free despite entering cardiac rehab. Figure 2 shows an EKG that was repeated post-operatively, along with images taken during the catheterization. 

## Discussion

The use of the internal mammary artery for revascularization of the LAD has been largely successful in part due to the rare occurrence of occlusive complications associated with IMA grafts. Indeed, in a study of 617 people, only 7.8% of those who had undergone LIMA revascularization had a stenosis of their graft between 50%-98%, while only 1.3% had a complete occlusion [4,5]. There are limited studies evaluating the causes of IMA graft failure as the success rate is so high, but we were able to find a few studies that evaluate this rare occurrence. When evaluating causes that lead to patients requiring revascularization of their IMA graft, kinking of the IMA graft during surgery was seen as a leading cause of stenosis of the graft [5]. Cases caused by kinking of the graft usually occur relatively soon after the procedure, which differs from our patient, who presented years after her procedure. Another study evaluating restenosis found that the degree of stenosis in the native vessel was a major predictor of IMA graft patency. Doppler studies illustrated that moderate stenosis, when compared to severe stenosis in the target vessel, was associated with the competitive flow that led to decreased anterograde flow in the arterial graft and, therefore could lead to failure of the graft [5]. This study also reported that when graft occlusion occurs, it is more likely to occur in LIMA compared to RIMA graft, further illustrating the rarity of our patient’s presentation.

Despite the relative infrequency of graft occlusion, our patient was found to have an occluded and atretic LIMA graft to the LAD about 11 years after her initial CABG surgery, and she underwent a redo bypass for the same single stenosis with RIMA to LAD. The cause of the LIMA occlusion was uncertain, but uncontrolled atherosclerosis was a possibility. Her native LAD was occluded at the time, making competitive flow and lack of LIMA-LAD flow unlikely to have been causative. About 16 months after her revision, she occluded her new arterial graft after appendicitis surgery, requiring a drug-eluting stent. We suspect that given the acute occlusion and eccentric and focal nature of the stenosis, her occlusion was atherosclerosis mediated. This, in the setting of her uncontrolled LDL-C, further aligns with our theory.

In regards to risk factors for IMA graft acute coronary syndromes, little is known given the rarity of the event, but some studies have attempted to explore this, largely in LIMA graft cases. Traditional risk factors, such as smoking, hypertension, obesity, and hyperlipidemia, which are usually linked to CAD, were evaluated in patients with LIMA graft stenosis to see if there is an association. In one study, LDL-C levels, smoking, and fasting plasma glucose levels were significantly associated with increased risk of LIMA graft stenosis [4], suggesting that atherosclerosis-while rare in IMA grafts-likely can play a role. It also appeared that attaining target LDL-C levels in patients who had stenosis of their graft was more difficult, which may imply that their hyperlipidemia was harder to control [5]. Our patient has severe dyslipidemia and has tried multiple lipid-lowering treatments with some, but not complete lipid improvement. This further supports our theory that her stenosis was related to her uncontrolled hyperlipidemia. Very few studies have evaluated RIMA graft occlusions, though we suspect the pathophysiology is similar.

For our patient, we adjusted her medications after her most recent admission by switching her from clopidogrel to ticagrelor 90 mg twice daily, given suspicion for the acute coronary syndrome (ACS) alongside tight control of her hyperlipidemia given suspicion for atherosclerotic mediated ACS. She was also started on losartan 12.5 mg daily and metoprolol succinate 50 mg daily, given her drop in left ventricular ejection fraction of less than 40%. She is being followed by a multidisciplinary team the following discharge.

## Conclusions

This case report illustrates how rare stenosis of an IMA graft is and aims to explore the different risk factors associated with such events. Traditional risk factors, especially smoking, hyperlipidemia, and diabetes, are significantly associated with stenosis of graft. Ultimately, though RIMA occlusion is rare, we suspect it was related to traditional risk factors in our patient. Therefore, controlling her dyslipidemia should be the top priority to prevent restenosis as well as to prevent any further cardiovascular events in her native or graft arteries.
